# Association Between Childhood Visual Acuity and Late Adolescent Psychotic Experiences: A Prospective Birth Cohort Study

**DOI:** 10.1093/schbul/sbab121

**Published:** 2021-10-08

**Authors:** Natalie Shoham, Joseph F Hayes, Claudia Cooper, Magnus Theodorsson, Gemma Lewis

**Affiliations:** 1 Division of Psychiatry, University College London, London, UK; 2 Camden and Islington NHS Foundation Trust, St Pancras Hospital, London, UK; 3 Department of Ophthalmology, King’s College Hospital, London, UK

**Keywords:** psychotic experiences, schizophrenia, visual acuity, visual impairment, ALSPAC

## Abstract

A cross-sectional association between visual impairment and psychosis exists, but longitudinal evidence from children and young people is limited. We investigated whether childhood visual acuity was associated with subsequent psychotic experiences. Our sample was 6686 individuals from the Avon Longitudinal Study of Parents and Children (ALSPAC). We investigated whether our primary exposures, best corrected visual acuity at ages 7 and 11, were associated with psychotic experiences at ages 17 and 24. We also tested whether the following exposures at ages 7 and 11 were associated with subsequent psychotic experiences: requiring glasses, presence of any visual impairment, and between-eye visual acuity difference; and at age 7: strabismus, measures of binocular vision, history of eye patch, near vision impairment, and abnormal saccadic or pursuit eye movements. Analyses used multilevel models before and after adjusting for confounders. Odds of psychotic experiences increased with each 0.1-point deterioration in visual acuity score at age 11 (adjusted odds ratio [AOR] 1.23; 95% confidence interval [CI] 1.06–1.42), and at age 7 (AOR 1.18; 95% CI 1.00–1.40). Wearing glasses and visual impairment at age 11 were associated with psychotic experiences (AOR 1.63; 95% CI 1.21–2.19; AOR 1.64; 95% CI 1.23–2.19, respectively). There was no evidence of an association with other visual exposures. Visual acuity impairment in childhood is associated with psychotic experiences in late adolescence. Future research should aim to elucidate the nature of this association.

## Introduction

Psychotic illnesses reduce quality of life and life expectancy.^1,2^ The point prevalence of schizophrenia is approximately 0.5%,^3^ whilst symptoms such as delusions and hallucinations affect 5%–6% of nonclinical populations.^4,5^ The peak incidence of both psychotic symptoms and diagnoses occurs in adolescence,^5–7^ which has recently been defined as ages 10–24 based on culturally extended periods of transitioning to adulthood.^8^

Visual impairment, typically defined as reduced visual acuity, has been proposed as a risk factor for psychotic symptoms and illnesses.^9,10^ In younger people the most common cause of reduced visual acuity is myopia,^11^ when light is focused too far in front of the retina causing blurred distance vision.^12^ Myopia affects at least 30% of people in Europe,^13^ but can often be corrected using lenses.^14^ Best corrected visual acuity refers to the highest acuity achievable with such aids.

The “Protection against Schizophrenia” (PaSZ) model proposes that aberrant visual input contributes to development of schizophrenia, and that optimal and lifelong absent vision protect against it.^9^ This is based on an absence of reported cases of a congenitally cortically blind person developing schizophrenia, despite shared risk factors.^15^ Silverstein et al have observed that compensatory higher-order processes in congenitally blind individuals enhance attention and auditory perceptual abilities in a reverse pattern to typical cognitive impairments seen in schizophrenia, potentially creating a buffering effect.^15^ Individuals whose vision deteriorates from normal lack these changes and may be susceptible to psychosis due to reliance on vision for cognitive processing.

Cross-sectional evidence shows that people with visual impairment are more likely to have psychosis than people without,^16^ but evidence regarding a temporal association is mixed.^17–21^ Furthermore, most studies focused on adults, although any prevention strategies might be most effective during neurodevelopment and before the peak incidence of first episode psychosis.^22^ Only 2, relatively small longitudinal studies were conducted with children. Visual dysfunction at ages 4 and 11–13 was associated with an increased risk of psychotic illness at ages 22 and 31–33, respectively.^23,24^ Both studies used composite exposure measures including squint and eye movement scores combined with acuity. It is therefore unclear which aspects of ocular function drove the association. The studies do not present results adjusted for confounders.

To our knowledge, this is the first large study investigating whether childhood visual impairment is associated with future psychotic experiences. We investigated whether poorer visual acuity at age 7 or 11 was associated with psychotic experiences at ages 17 and 24. We also assessed, as secondary exposures: tests of binocular vision, near vision impairment, and eye movements; in line with other studies.^23–25^

## Methods

We published our advance protocol on *protocols.io.*^26^

### Sample

The Avon Longitudinal Study of Parents and Children (ALSPAC) is an ongoing UK birth cohort designed to further knowledge of determinants of illness and health.^27,28^ All pregnant women in the catchment area with a due date between April 1, 1991 and December 31, 1992 were eligible for invitation at antenatal appointments and through advertisements, and 14 541 were originally recruited.^27,28^ Their children were followed into adulthood. At age 7, 913 additional children were enrolled. In total, 14 901 infants surviving to age 1 were included.^29^ The ALSPAC Law and Ethics Committee and Local Research Ethics Committees provided ethical approval for the study and participants gave written informed consent. Study data were collected and managed using the REDCap electronic data capture tool hosted at the University of Bristol: a secure, web-based software platform.^30^

Further details of the cohort profile and a fully searchable data dictionary can be obtained through the study website (https://www.bristol.ac.uk/alspac/).

### Outcome Variable

The Psychotic-Like Symptoms Screening Interview (PLIKSi) is a semistructured interview based on the Schedule for Clinical Assessment in Neuropsychiatry (SCAN),^31^ designed to assess psychotic experiences in nonclinical populations.^32^ It includes 11 core questions about psychotic experiences (hallucinations, delusions, and thought interference).^32^ The PLIKSi has been validated in ALSPAC and was administered by trained interviewers.^32^ Our primary outcome measures were psychotic experiences at age 17 or 24 as binary variables (suspected or definite psychotic experiences/none). We did not include symptoms occurring only in partial sleep states or fever, consistent with standard use of the PLIKSi.^33^

### Exposure Variables

Trained orthoptists performed visual assessments at ages 7 and 11. Visual acuity was measured using the Early Treatment Diabetic Retinopathy Study (ETDRS) chart to give a Logarithm of Minimal Angle of Resolution (LogMAR) score ranging from −0.3 to 1.0.^34^ Zero is equivalent to “normal” 6/6 or 20/20 vision, with negative numbers indicating “better than normal” and positive numbers indicating “worse than normal” vision.^35^ Carers reported whether children wore glasses. Children who had used glasses in the preceding 6 months wore these, and carers supported with matching cards if the child did not know the alphabet. Testing was performed using patches to occlude 1 eye and repeated using a pinhole; a simple device to improve acuity in refractive errors including myopia.^35^ Best corrected visual acuity was the better measurement; with or without the pinhole.

Additional measures at age 7 included: presence of manifest strabismus (consistent squint)^35^ on cover/uncover test; abnormal binocular fusion (ability to fuse images from each eye),^36^ determined by prism cover and Worth Four Dots tests; history of eye patch reported by parent or carer; autorefraction results; and abnormal pursuit (smooth) and saccadic (rapid) eye movements as observed by the examiner using a picture target and light.^37^ The autorefractor estimates errors in focusing of light on the retina in Dioptres; a measure of lens strength required for correction, with positive numbers indicating near visual impairment.^38,39^

Our primary exposure variables were best corrected LogMAR scores at ages 7 and 11 as continuous variables, as the most informative measure of overall visual ability. We tested between-eye visual acuity difference at each age as secondary continuous exposure variables.^20^ We created a binary variable to compare types of vision: normal vision (LogMAR ≤0) without glasses, or visual impairment (reduced vision; or normal vision with glasses). We also analyzed as binary exposure variables: glasses use in past 6 months (yes/no) at ages 7 and 11; and at age 7, presence of manifest strabismus; history of patch (as a proxy for amblyopia or “lazy eye”)^35^; abnormal prism test; abnormal Worth Four Dots test; near vision impairment (≥+2.00 Dioptres in either eye on autorefraction); abnormal pursuit movements; and abnormal saccadic eye movements.

### Putative Confounding Variables

Confounding variables were selected a priori based on available literature^5,33,40–49^ and included in final models if missing data did not preclude complete case model analyses.

We included sex of child and socioeconomic status of mother, but not ethnicity of parents, owing to small numbers in all ethnic groups except White British (96%).^27^

Tobacco use and infection in pregnancy were established by questionnaires administered to expectant mothers, and we assessed these as binary variables (present/absent). We included maternal vitamin D consumption estimated from a dietary questionnaire at 32 weeks’ gestation as a continuous variable and number of previous pregnancies reported as a discrete variable.

We included Intelligence Quotient (IQ) score at age 8 as a continuous variable, only in analyses when the exposure was measured subsequently (at age 11). We used parental education, a categorical variable for each of mother and mother’s partner (CSE/O level/A level/vocational/degree).

The Strength and Difficulties Questionnaire (SDQ) is a measure of childhood adjustment and psychopathology.^50^ Parents completed this when children were aged 6–7, and we included it as a continuous measure of baseline psychopathology. We included maternal Edinburgh Postnatal Depression Scale (EPDS) score when children were 2–3 years old.^51^

### Analysis

#### Descriptive Statistics

We calculated numbers and percentages for categorical variables. We report mean and standard deviation for continuous variables that appeared normally distributed on visual inspection, and median and interquartile range for skewed variables.

#### Missing Data

**Fig. 1.**
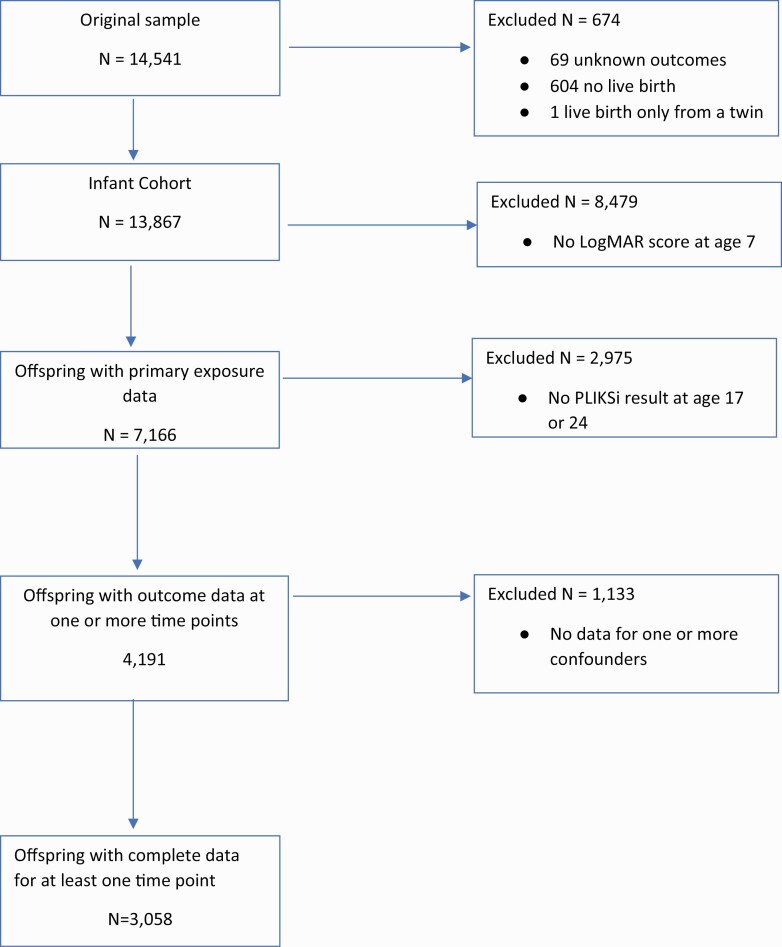
Missing data flowchart. *Note*: LogMAR, Logarithm of Minimal Angle of Resolution; N, number of participants; PLIKSi, Psychotic-Like Experiences Symptoms Interview.

Some participants dropped out and others missed assessments intermittently (figure 1). To maximize the sample size and reduce attrition bias, our main analyses used a multiply imputed dataset of participants with a LogMAR score at age 7 and at least 1 of 10 available short Mood and Feelings Questionnaire (sMFQ) scores taken from ages 9 through to 22. The sMFQ is a 13-item self-report measure of depressive symptoms^52^ which is associated with the PLIKSi in the ALSPAC cohort and improves prediction of missing PLIKSi values.^53^ For example, for the sMFQ score with least missing data (at age 9), the odds of scoring positive on the PLIKSi were increased by 11% for each additional point (odds ratio [OR] 1.11, 95% confidence interval [CI] 1.07–1.16).

We used chained equations through the command *mi impute chained* in STATA version 16.0^54^ to generate the imputed dataset, with 100 imputations, including all exposure, confounder, and outcome variables. This process uses a “burn-in” of 10 cycles for each imputation. We imputed binary variables using logistic regression, ordinal variables using ordered logistic regression, and continuous variables using linear regression. Where continuous variables were nonnormally distributed, we used progressive mean matching via the *pmm* command to impute only observed values, making the distribution of imputed variables consistent with the observed dataset.^55^

Multiple imputation (MI) relies on the assumption that data are Missing At Random (MAR): ie, that missing values are associated with variables observed in the dataset. The latter assumption was made more likely through inclusion of auxiliary variables (variables associated with missing data but not included in our main analyses), such as mother’s housing situation, family income, conduct disorder score, and mother’s marital status, which are associated with missingness in the ALSPAC data.^53,56^

#### Primary Analyses

We used multilevel logistic regression (on the multiply imputed dataset) in *Stata version 16.0*.^54^ Imputed data were combined following Rubin’s rules,^55^ producing an OR for scoring positive on the PLIKSi at age 17 or 24 for each 0.1-point deterioration in LogMAR score. Our model accounted for within-individual correlation between PLIKSi scores at age 17 or 24 using a random intercept for each individual. We repeated this analysis using each exposure; unadjusted and adjusted for putative confounding variables. We did not use Bonferroni corrections because multiple exposures were testing the same a priori hypothesis, and Bonferroni tests increase the chance of a type II error without significant benefit in this situation.^57^

#### Sensitivity Analyses

We report results from the complete case sample for comparison.

Additionally, visual hallucinations directly secondary to reduced vision in Charles Bonnet syndrome are a well-recognized phenomenon, and generally considered distinct from other psychotic experiences.^58^ We repeated our primary analyses in the complete case sample excluding individuals who reported visual hallucinations except in states of fever, intoxication, or sleep states, to assess whether these drove any association.

## Results

### Description of the Sample

Characteristics of participants according to visual acuity (“normal” vision [LogMAR ≤0] and reduced vision [LogMAR >0]) are shown in table 1.

**Table 1.** Characteristics of Sample With Complete Data for Visual Acuity at Age 7

Characteristic Total *N* = 7166	Whole Sample *N* (%)	Group With LogMAR Score ≤0 *N* (%)	Group With LogMAR Score >0 *N* (%)
Male	3584 (50.1)	3135 (50.3)	449 (48.9)
Maternal socioeconomic status based on occupation			
Professional	248 (4.2)	228 (4.5)	20 (2.7)
Managerial and technical	1871 (32.0)	1661 (32.5)	210 (28.6)
Skilled nonmanual	2512 (43.0)	2184 (42.7)	328 (44.7)
Skilled manual	205 (3.5)	175 (3.4)	30 (4.1)
Partly skilled	849 (14.5)	728 (14.3)	121 (16.5)
Unskilled	159 (2.7)	134 (2.6)	25 (3.4)
Maternal educational level			
CSE	839 (12.9)	689 (12.1)	150 (18.3)
Vocational	546 (8.4)	464 (8.2)	82 (10.0)
O level	2337 (35.9)	2045 (40.0)	292 (35.7)
A level	1733 (26.6)	1532 (26.9)	201 (24.5)
Degree	1052 (16.2)	958 (16.8)	94 (11.5)
Mother′s partner′s educational level			
CSE	1229 (19.4)	1009 (18.3)	220 (27.4)
Vocational	493 (7.8)	430 (7.8)	63 (7.8)
O level	1439 (22.7)	1245 (22.5)	194 (24.1)
A level	1767 (27.9)	1577 (28.5)	190 (23.6)
Degree	1402 (22.2)	1265 (22.9)	137 (17.0)
Infection during first trimester of pregnancy	1467 (23.6)	1269 (23.4)	198 (25.2)
Maternal smoking in pregnancy	1248 (18.8)	1068 (18.5)	180 (21.3)
Mother′s parity in pregnancy	Median 1 IQR 0–1	Median 1 IQR 0–1	Median 1 Range 0–8
IQ aged 8	Mean 105.2 SD 16.1	Mean 105.9 SD 15.7	99.6 (17.5)
SDQ score aged 81 months	Median 6 IQR 4–10	Median 6 IQR 4–10	Median 7 IQR 4–11
Maternal EPDS score in pregnancy	Median 6 IQR 3–9	Median 6 IQR 3–9	Median 6 IQR 3–10
Maternal vitamin D consumption in pregnancy in micrograms	Median 3.5 IQR 2.5–5.4	Median 3.5 IQR 2.5–5.4	Median 3.3 IQR 2.3–5.1
LogMAR score aged 7	Mean −0.06 SD 0.07		
LogMAR score aged 11	Mean −0.15 SD (0.09)	Mean −0.16 SD 0.07	Mean −0.06 SD 0.1
Needed glasses aged 7	754 (10.6)	452 (7.3)	302 (32.9)
Needed glasses aged 11	989 (18.2)	691 (14.5)	298 (44.4)
Visual impairment (LogMAR >0 or needing glasses) at age 7	1370 (19.2)		
Visual impairment (LogMAR >0 or needing glasses) at age 11	1030 (19.4)	708 (15.2)	322 (49.5)
Manifest strabismus aged 7	144 (2.0)	81 (1.3)	63 (6.9)
Abnormal prism test aged 7	746 (10.5)	612 (9.8)	134 (14.6)
History of eye patch aged 7	226 (3.2)	143 (2.3)	83 (9.0)
Abnormal Worth′s Four Dots test aged 7	209 (3.8)	129 (2.7)	80 (12.0)
Impaired near vision aged 7	278 (3.9)	149 (2.4)	129 (14.1)
Abnormal saccadic eye movements aged 7	361 (5.8)	293 (5.4)	68 (8.6)
Abnormal smooth pursuit eye movements aged 7	522 (7.3)	433 (6.9)	89 (9.7)
Scored positive on PLIKSi aged 17	261 (7.4)	228 (7.2)	33 (8.3)
Scored positive on PLIKSi aged 24	291 (10.1)	254 (9.9)	37 (11.4)
Scored positive on PLIKSi at either age	481 (11.5)	419 (11.3)	62 (12.6)

*Note*: EPDS, Edinburgh Postnatal Depression Scale; IQ, Intelligence Quotient; IQR, interquartile range; LogMAR, Logarithm of Minimal Angle of Resolution, where 0 = “normal” vision, <0 = “better than normal” vision, and >0 = reduced vision; PLIKSi, Psychotic-Like Experiences Symptoms Interview; SDQ, Strengths and Difficulties Questionnaire. Missing data (%): sex (0.2); maternal ethnicity (9.5); maternal socioeconomic status (18.5); maternal educational level (9.2); mother’s partner’s educational level (11.7); infection during first trimester of pregnancy (13.3); maternal smoking in pregnancy (7.4); mother’s parity in pregnancy (8.8); IQ aged 8 (18.0); SDQ score aged 81 months (20.3); maternal EPDS score in pregnancy (13.2); maternal vitamin D consumption in pregnancy (11.3); glasses use aged 7 (0.03); glasses use aged 11 (24.3); visual impairment aged 7 (0.3); visual impairment aged 11 (25.9); manifest strabismus aged (0.1); prism test aged 7 (0.4); Worth’s Four Dots test aged 7 (23.1); impaired near vision aged 7 (0.3); saccadic eye movements aged 7 (12.5); pursuit eye movements aged 7 (0.1); PLIKSi aged 17 (50.4); PLIKSi aged 24 (59.7); no PLIKSi result at either age (41.5).

Six thousand six hundred and eighty-six individuals provided visual acuity scores aged 7 and at least 1 MFQ score. These individuals comprised the multiply imputed sample in primary analyses. Complete data on the primary exposure at age 7, all confounders, and outcome data at one or both of ages 17 and 24 were available for 3058 individuals. These individuals comprised the complete case sample. Differences between participants with and without missing data (ie, those in the imputed and complete case samples) are shown in supplementary table 1.

From the sample with complete visual acuity data at age 7, 481 (11.5%) individuals with primary exposure data scored positive on the PLIKSi at 1 or more time points; 261 (7.4%) at age 17 and 291 (10.1%) at age 24.

Potential confounders measured but not included due to a high proportion of missing data (%) included: resuscitation at birth (46), gestational age at birth (44), and polygenic risk score for schizophrenia (28). Bivariable models in the sample with all available data suggested that these were not significant confounders, however.

### Primary Results

In the MI analysis, we found evidence that the odds of adolescent psychotic experiences increased with each 0.1-point deterioration in LogMAR score at age 7: OR 1.26 (95% CI 1.06–1.49), and at age 11: OR 1.31 (95% CI 1.13–1.51) (table 2). Evidence of these associations attenuated but remained after adjustments; at age 7: adjusted odds ratio (AOR) 1.18 (95% CI 1.00–1.40), and at age 11: AOR 1.23 (95% CI 1.06–1.42). Visual inspection of histograms suggested that greater variation in the exposure could account for the stronger evidence of association at age 11 relative to age 7.

**Table 2.** Odds of Scoring Positive on Psychotic-Like Symptoms Interview (PLIKSi) According to Eyesight Variables in Multiply Imputed Data

Exposure	N	OR (95% CI)	*P* Value	AOR (95% CI)^c^	*P* Value
Outcome: positive result on PLIKSi aged 24 or aged 17					
Best corrected visual acuity aged 7^a^	6686	1.26 (1.06–1.49)	.008*	1.18 (1.00–1.40)	.057
Best corrected visual acuity aged 11^a^	6686	1.31 (1.13–1.51)	<.001*	1.23 (1.06–1.42)	.006*
Difference in acuity between eyes aged 7^a^	6686	1.05 (0.88–1.25)	.565	1.03 (0.86–1.22)	.782
Difference in acuity between eyes aged 11^a^	6686	0.94 (0.70–1.25)	.654	0.92 (0.69–1.23)	.571
Child needed glasses aged 7	6686	1.56 (1.02–2.38)	.039*	1.42 (0.93–2.17)	.103
Child needed glasses aged 11	6686	1.73 (1.28–2.33)	<.001*	1.63 (1.21–2.19)	.001*
Normal vision with glasses or subnormal vision aged 7^b^	6686	1.41 (1.03–1.92)	.032	1.28 (0.93–1.75)	.125
Normal vision with glasses or subnormal vision aged 11^b^	6686	1.75 (1.31–2.34)	<.001*	1.64 (1.23–2.19)	.001*
Manifest strabismus aged 7	6686	0.56 (0.19–1.65)	.292	0.46 (0.15–1.37)	.161
Abnormal prism test aged 7	6686	1.25 (0.83–1.89)	.290	1.20 (0.79–1.82)	.389
History of eye patch aged 7	6686	1.07 (0.54–2.12)	.841	0.97 (0.49–1.92)	.936
Abnormal Worth Four Dots test aged 7	6686	0.85 (0.39–1.88)	.693	0.79 (0.36–1.74	.555
Impaired near vision aged 7	6686	0.62 (0.29–1.31)	.209	0.57 (0.27–1.21)	.140
Abnormal saccadic eye movements aged 7	6686	0.82 (0.45–1.49)	.521	0.73 (0.40–1.33)	.300
Abnormal pursuit eye movements aged 7	6686	0.68 (0.40–1.18)	.169	0.64 (0.37–1.11)	.112

*Note*: 95% CI, 95% confidence interval; AOR, adjusted odds ratio; IQ, Intelligence Quotient; *N*, number of individuals in analysis; OR, odds ratio. At age 11, this was further adjusted for IQ aged 8.

^a^Per 0.1 point deterioration.

^b^Relative to group with normal vision without glasses.

^c^Adjusted for sex; mother’s socioeconomic status; educational level of mother and mother’s partner; maternal smoking during pregnancy; perinatal infection during first trimester; parity of mother during pregnancy; mother’s reported vitamin D intake during pregnancy; Strengths and Difficulties Questionnaire (SDQ) score aged 81 months; and maternal Edinburgh Postnatal Depression Scale (EPDS) score in pregnancy.

*P < .05.

### Secondary Analyses

Following adjustment there was evidence of association between greater odds of adult psychotic experiences and needing glasses (AOR 1.63, 95% CI 1.21–2.19); and any visual impairment (LogMAR >0 or requiring glasses) (AOR 1.64, 95% CI 1.23–2.19), at age 11 (table 2). AORs were also suggestive of a positive association at age 7, but statistical evidence was weaker.

There was no evidence of an association with the outcome for between-eye visual acuity difference at either age, or with manifest strabismus, abnormal prism test, history of eye patch, abnormal Worth Four Dots test, impaired near vision, or abnormal saccadic or pursuit eye movements at age 7, either before or after adjustment.

### Complete Case Sample

When we repeated the analyses in the complete case sample, estimates for the association between psychotic experiences and LogMAR scores, glasses use, and visual impairment were similar to MI analyses, with CIs largely overlapping (table 3). Unexpectedly, there was weak evidence of a negative association between manifest strabismus (AOR 0.23, 95% CI 0.06–0.91), abnormal saccadic eye movements (AOR 0.44, 95% CI 0.20–0.98), and abnormal pursuit eye movements (AOR 0.47, 95% CI 0.24–0.90) at age 7 and psychotic experiences, that was not seen in MI analyses.

**Table 3.** Odds of Scoring Positive on Psychotic-Like Symptoms Interview (PLIKSi) According to Eyesight Variables in Complete Case Sample

Exposure	*N*	OR (95% CI)	*P* Value	AOR (95% CI)^c^	*P* Value
Outcome: positive result on PLIKSi aged 24 or aged 17					
Best corrected visual acuity aged 7^a^	3058	1.37 (1.08–1.74)	.009*	1.29 (1.02–1.64)	.037*
Best corrected visual acuity aged 11^a^	3074	1.23 (1.01–1.50)	.037*	1.16 (0.94–1.42)	.163
Difference in acuity between eyes aged 7^a^	3058	0.93 (0.70–1.24)	.628	0.90 (0.68–1.21)	.498
Difference in acuity between eyes aged 11^a^	3074	0.98 (0.67–1.43)	.919	0.97 (0.66–1.43)	.886
Child needed glasses aged 7	3379	1.27 (0.78–2.06)	.329	1.16 (0.71–1.88)	.552
Child needed glasses aged 11	3138	1.97 (1.32–2.94)	.001*	1.91 (1.28–2.85)	.002*
Normal vision with glasses or subnormal vision aged 7^b^	3051	1.36 (0.89–2.08)	.151	1.20 (0.79–1.85)	.393
Normal vision with glasses or subnormal vision aged 11^b^	3073	2.09 (1.40–3.11)	<.001*	1.99 (1.33–2.97)	.001*
Manifest strabismus aged 7	3382	0.28 (0.07–1.07)	.064	0.23 (0.06–0.91)	.037*
History of eye patch aged 7	3387	0.50 (0.19–1.29)	.150	0.42 (0.16–1.10)	.077
Abnormal prism test aged 7	3373	0.88 (0.53–1.44)	.606	0.83 (0.50–1.37)	.456
Abnormal Worth Four Dots test aged 7	2556	0.64 (0.23–1.78)	.392	0.55 (0.20–1.51)	.243
Impaired near vision aged 7	3368	0.56 (0.22–1.39)	.207	0.50 (0.20–1.26)	.141
Abnormal saccadic eye movements aged 7	2925	0.51 (0.24–1.12)	.092	0.44 (0.20–0.98)	.044*
Abnormal pursuit eye movements aged 7	3380	0.46 (0.24–0.89)	.020	0.47 (0.24–0.90)	.023*

*Note*: 95% CI, 95% confidence interval; AOR, adjusted odds ratio; IQ, Intelligence Quotient; *N*, number of individuals in analysis; OR, odds ratio. At age 11, this was further adjusted for IQ aged 8.

^a^Per 1 point deterioration.

^b^Relative to group with normal vision without glasses.

^c^Adjusted for sex; mother’s socioeconomic status; educational level of mother and mother’s partner; maternal smoking during pregnancy; perinatal infection during first trimester; parity of mother during pregnancy; mother’s reported vitamin D intake during pregnancy; Strengths and Difficulties Questionnaire (SDQ) score aged 81 months; and maternal Edinburgh Postnatal Depression Scale (EPDS) score in pregnancy.

*P < .05.

### Sensitivity Analysis

Excluding outcome measures from individuals who reported visual hallucinations at each time point did not weaken the evidence of associations in the complete case sample (supplementary table 2).

## Discussion

### Main Findings

There are 2 plausible explanations for our findings that best corrected visual acuity in childhood; needing glasses; and any visual impairment aged 11 are associated with future psychotic experiences. First, reduced childhood visual acuity may be a risk factor for these experiences. Our findings are consistent with the PaSZ model, and previous work proposing this.^9,15^ We included no known blind participants, so can comment on the first PaSZ model assertion, that visual impairment is associated with an increased risk of psychosis; but not the second, that absent vision is protective. Alternatively, our findings could be explained by early life central nervous system dysfunction predisposing to both visual impairment and psychosis.

Although we found evidence that glasses use and visual impairment at age 11 are associated with psychotic experiences, there was very weak evidence for these exposures at age 7. Best corrected visual acuity improved overall at age 11 compared to age 7 with a slightly broader distribution of values, suggesting that we were predominantly assessing differences within the “normal” range. In our multiply imputed dataset, more children wore glasses at age 11, allowing greater power to detect the association with glasses use. The increase in corrected myopia prevalence is expected since a process of ocular elongation occurs in infants and may continue until adulthood.^14^

The finding that eye movement abnormalities and squint were not associated with psychotic experiences seems surprising, given that these are some of the most widely replicated neurological abnormalities in schizophrenia.^59^ This might be because these measures are associated with psychotic illnesses but not other types of psychotic experience, or because they occur closer to the time when psychotic experiences are established, or result from these.

### Strengths and Limitations

To our knowledge, this is the first large study to assess the association between reduced childhood visual acuity and psychotic experiences in adolescence. Strengths include the use of a large birth cohort, and the ability to consider a wide range of potential confounders. The inclusion of children at age 7 is particularly helpful in the context of vision. The resulting age range represents the widest consensus as to when the visual pathway is reaching its final stages of development. Therefore, the development of amblyopia (diminished vision secondary to impaired visual stimulation during this period) would most likely have manifested in this sample of children, and the subsequent measure of reduced visual acuity would have been identified and included in analysis.^60^

Several limitations should be noted. The ASLPAC cohort consists mostly of White British participants,^61^ and cannot be considered fully representative of the population of the UK or global community. Although we aimed to include a wide range of confounding variables, residual and unmeasured confounding cannot be eliminated. The proportion of attrition and missing data in ALSPAC is also substantial and could bias findings. In our sample, 57% of participants with complete primary exposure data aged 7 were missing data in the confounders or outcome. The negative association with strabismus and eye movements seen in the complete case sample suggests bias caused by missing data. We have aimed to mitigate this by using MI; simulations in the ALSPAC dataset show that even when outcome data are Missing Not At Random (MNAR), use of MI with appropriate auxiliary variables gives less biased results compared to those from complete case analyses.^62^

Although visual acuity was measured objectively, full engagement with the process was required, and children’s’ motivation may have influenced test results. Reducing visual acuity to a binary measure reduces its sensitivity, and even small differences within the normal range of vision can lead to significant problems in visual processing. However, we still found associations.^63^ We could not assess uncorrected acuity, which likely led to an underestimate of the true strength of associations. Conversely, the PLIKSi relies on self-reporting, and it is possible that psychotic experiences were under-detected due to stigma, which might have weakened ability to detect an association.

Although we have shown an association between visual acuity and psychotic experiences, this does not necessarily equate to an association with psychotic illnesses, since these phenomena do not entirely overlap.^5^ Psychotic experiences are associated with a range of psychiatric morbidity,^5^ and so our findings could be driven by an association between visual impairment common mental disorder. Even so, the PaSZ model describes a gradient of risk of psychotic symptomatology as a continuous phenomenon according to degree of visual capacity, and should therefore be generalizable to a broad range of psychotic experiences.^9^

We have not explored possible mediators or effect modifiers of the association in this study. Confounders such as shared genetic mechanisms or risk factors such as birth trauma could explain the association.^64^ It is also possible that subsequent environmental influences, such as bullying or trauma, might lie on a causal pathway between visual impairment and psychosis.^65^

### Comparison With Other Literature

Our findings extend those from 2 small cohort studies of children which found that ocular deficits predicted adulthood diagnosis of schizophrenia.^23,24^ We have demonstrated this association in a large sample, using psychotic experiences rather than diagnoses. We have found that visual acuity impairment specifically, rather than other ocular measures, appears to account for the association. This might be because visual acuity impairments affect more people, leading to greater power. Alternatively, it might be because acuity deficits emerge earlier in the development of central nervous system dysfunction than do other ocular deficits. Our results are consistent with those from a Swedish cohort study, which showed that poorer visual acuity at ages 18–19 was associated with subsequent diagnosis of psychotic illnesses.^20^ The Swedish study also found that between-eye visual acuity difference was associated with psychotic illness. We did not replicate this finding: perhaps because we measured corrected rather than uncorrected visual acuity, so were less likely to detect between-eye differences; or because this association did not exist in our sample, which was younger; or because we explore psychotic experiences rather than illnesses.^20^ The Swedish study is one of the 2 largest cohort studies in older adolescents on this topic. The second, Israeli study gave conflicting findings despite reporting on a comparable population of young army conscripts aged 16–17. It reported that refractive errors in late adolescence were negatively associated with subsequent schizophrenia.^21^ It did not describe how refractive error was defined, so perhaps only severe visual impairment was counted as an exposure, which could explain the discrepancy both with our study and the Swedish study.

A 2020 systematic review collated studies that used optical coherence tomography and electroretinography (ERG) to compare ophthalmic structure and function in people with schizophrenia and people without.^66^ Across studies, there is evidence of retinal thinning and altered retinal waveforms in schizophrenia, which are conceptualized as biomarkers representing an underlying neuropathological process.^66^ The timing of the development of these alterations is unknown, but rates of ERG abnormalities are also elevated in children at high risk of psychotic illness.^67^ Reduced visual acuity could therefore result from this process in some children in our study and share an underlying neuropathology or genetic predisposition with psychosis.

## Conclusions

Our findings support a temporal association whereby childhood visual acuity impairment is associated with late adolescent psychotic experiences. Future research should aim to elucidate the underlying mechanisms behind this association, to further our understanding of the development of psychotic illnesses and symptoms and how they relate to vision.

## Supplementary Material

sbab121_suppl_Supplementary_Tables

## References

[1] Hjorthøj C , Stürup AE, McGrath JJ, Nordentoft M. Years of potential life lost and life expectancy in schizophrenia: a systematic review and meta-analysis. Lancet Psychiatry. 2017;4(4):295–301. 28237639 10.1016/S2215-0366(17)30078-0

[2] Fleischhacker WW , Arango C, Arteel P, et al. Schizophrenia—time to commit to policy change. Schizophr Bull. 2014;40(suppl 3):S165–S194. 24778411 10.1093/schbul/sbu006 PMC4002061

[3] Saha S , Chant D, Welham J, McGrath J. A systematic review of the prevalence of schizophrenia. PLoS Med. 2005;2(5):e141. 15916472 10.1371/journal.pmed.0020141 PMC1140952

[4] Shoham N , Cooper C, Lewis G, Bebbington P, McManus S. Temporal trends in psychotic symptoms: repeated cross-sectional surveys of the population in England 2000–14. Schizophr Res. 2021;228:97–102. 33434740 10.1016/j.schres.2020.11.057

[5] Bourgin J , Tebeka S, Mallet J, Mazer N, Dubertret C, Le Strat Y. Prevalence and correlates of psychotic-like experiences in the general population. Schizophr Res. 2020;215:371–377. 31477372 10.1016/j.schres.2019.08.024

[6] Zammit S , Horwood J, Thompson A, et al. Investigating if psychosis-like symptoms (PLIKS) are associated with family history of schizophrenia or paternal age in the ALSPAC birth cohort. Schizophr Res. 2008;104(1–3):279–286. 18562177 10.1016/j.schres.2008.04.036

[7] Kirkbride JB , Errazuriz A, Croudace TJ, et al. Incidence of schizophrenia and other psychoses in England, 1950–2009: a systematic review and meta-analyses. PLoS One. 2012;7(3):e31660. 22457710 10.1371/journal.pone.0031660 PMC3310436

[8] Sawyer SM , Azzopardi PS, Wickremarathne D, Patton GC. The age of adolescence. Lancet Child Adolesc Health. 2018;2(3):223–228. 30169257 10.1016/S2352-4642(18)30022-1

[9] Landgraf S , Osterheider M. “To see or not to see: that is the question.” The “Protection-Against-Schizophrenia” (PaSZ) model: evidence from congenital blindness and visuo-cognitive aberrations. Front Psychol. 2013;4:352. 23847557 10.3389/fpsyg.2013.00352 PMC3696841

[10] Royal National Institute for the Blind. *The Criteria for Certification*. https://www.rnib.org.uk/eye-health/registering-your-sight-loss/criteria-certification. Accessed January 6, 2021.

[11] McCarty CA , Taylor HR. Myopia and vision 2020. Am J Ophthalmol. 2000;129(4):525–527. 10764864 10.1016/s0002-9394(99)00444-4

[12] Cooper J , Tkatchenko AV. A review of current concepts of the etiology and treatment of myopia. Eye Contact Lens. 2018;44(4):231–247. 29901472 10.1097/ICL.0000000000000499 PMC6023584

[13] Williams KM , Verhoeven VJ, Cumberland P, et al. Prevalence of refractive error in Europe: the European Eye Epidemiology (E(3)) Consortium. Eur J Epidemiol. 2015;30(4):305–315. 25784363 10.1007/s10654-015-0010-0 PMC4385146

[14] Morgan IG , Ohno-Matsui K, Saw SM. Myopia. Lancet. 2012;379(9827):1739–1748. 22559900 10.1016/S0140-6736(12)60272-4

[15] Silverstein SM , Wang Y, Keane BP. Cognitive and neuroplasticity mechanisms by which congenital or early blindness may confer a protective effect against schizophrenia. Front Psychol. 2012;3:624. 23349646 10.3389/fpsyg.2012.00624 PMC3552473

[16] Shoham N , Eskinazi M, Hayes JF, Lewis G, Theodorsson M, Cooper C. Associations between psychosis and visual acuity impairment: a systematic review and meta-analysis. Acta Psychiatr Scand. 2021:6–27. 10.1111/acps.13330 PMC8504204 34028803

[17] Stafford J , Howard R, Dalman C, Kirkbride JB. The incidence of nonaffective, nonorganic psychotic disorders in older people: a population-based cohort study of 3 million people in Sweden. Schizophr Bull. 2019;45(5):1152–1160. 30339239 10.1093/schbul/sby147 PMC6737541

[18] Hamedani AG , Thibault DP, Shea JA, Willis AW. Self-reported vision and hallucinations in older adults: results from two longitudinal US health surveys. Age Ageing. 2020;49(5):843–849. 32253434 10.1093/ageing/afaa043 PMC7444669

[19] Blazer DG , Hays JC, Salive ME. Factors associated with paranoid symptoms in a community sample of older adults. Gerontologist. 1996;36(1):70–75. 8932412 10.1093/geront/36.1.70

[20] Hayes JF , Picot S, Osborn DPJ, Lewis G, Dalman C, Lundin A. Visual acuity in late adolescence and future psychosis risk in a cohort of 1 million men. Schizophr Bull. 2018;45(3):571–578. 10.1093/schbul/sby084 PMC6483575 29901774

[21] Caspi A , Vishne T, Reichenberg A, et al. Refractive errors and schizophrenia. Schizophr Res. 2009;107(2-3):238–241. 19019632 10.1016/j.schres.2008.09.022

[22] Giedd JN , Blumenthal J, Jeffries NO, et al. Brain development during childhood and adolescence: a longitudinal MRI study. Nat Neurosci. 1999;2(10):861–863. 10491603 10.1038/13158

[23] Schubert E , Henriksson K, McNeil T. A prospective study of offspring of women with psychosis: visual dysfunction in early childhood predicts schizophrenia-spectrum disorders in adulthood. Acta Psychiatr Scand. 2005;112(5):385–393. 16223427 10.1111/j.1600-0447.2005.00584.x

[24] Schiffman J , Maeda JA, Hayashi K, et al. Premorbid childhood ocular alignment abnormalities and adult schizophrenia-spectrum disorder. Schizophr Res. 2006;81(2–3):253–260. 16242918 10.1016/j.schres.2005.08.008

[25] Shoham N , Lewis G, Hayes J, et al. Psychotic symptoms and sensory impairment: findings from the 2014 adult psychiatric morbidity survey. Schizophr Res. 2020;215:357–364. 31481335 10.1016/j.schres.2019.08.028 PMC7613093

[26] Shoham N , Hayes JF, Lewis G, Cooper C. *Protocol: Association Between Visual Acuity and Subsequent Psychotic-Like Experiences in the Avon Longitudinal Study of Parents and Children*. 2021. https://www.protocols.io/view/protocol-association-between-visual-acuity-and-sub-btvdnn26. Accessed April 9, 2021.

[27] Boyd A , Golding J, Macleod J, et al. Cohort profile: the “Children of the 90s”—the index offspring of the Avon Longitudinal Study of Parents and Children. Int J Epidemiol. 2013;42(1):111–127. 22507743 10.1093/ije/dys064 PMC3600618

[28] Fraser A , Macdonald-Wallis C, Tilling K, et al. Cohort profile: the Avon Longitudinal Study of Parents and Children: ALSPAC mothers cohort. Int J Epidemiol. 2013;42(1):97–110. 22507742 10.1093/ije/dys066 PMC3600619

[29] Lewis G , Button KS, Pearson RM, Munafò MR, Lewis G. Inhibitory control of positive and negative information and adolescent depressive symptoms: a population-based cohort study. Psychol Med. 2020:1–11. 10.1017/S0033291720002469 32677595

[30] Harris PA , Taylor R, Thielke R, Payne J, Gonzalez N, Conde JG. Research electronic data capture (REDCap)—a metadata-driven methodology and workflow process for providing translational research informatics support. J Biomed Inform. 2009;42(2):377–381. 18929686 10.1016/j.jbi.2008.08.010 PMC2700030

[31] Wing JK , Babor T, Brugha T, et al. SCAN. Schedules for Clinical Assessment in Neuropsychiatry. Arch Gen Psychiatry. 1990;47(6):589–593. 2190539 10.1001/archpsyc.1990.01810180089012

[32] Zammit S , Kounali D, Cannon M, et al. Psychotic experiences and psychotic disorders at age 18 in relation to psychotic experiences at age 12 in a longitudinal population-based cohort study. Am J Psychiatry. 2013;170(7):742–750. 23639948 10.1176/appi.ajp.2013.12060768

[33] Zammit S , Thomas K, Thompson A, et al. Maternal tobacco, cannabis and alcohol use during pregnancy and risk of adolescent psychotic symptoms in offspring. Br J Psychiatry. 2009;195(4):294–300. 19794196 10.1192/bjp.bp.108.062471

[34] Rosser DA , Laidlaw DA, Murdoch IE. The development of a “reduced logMAR” visual acuity chart for use in routine clinical practice. Br J Ophthalmol. 2001;85(4):432–436. 11264133 10.1136/bjo.85.4.432 PMC1723918

[35] Olver J , Cassidy L, Jutley G, Crawley L. Ophthalmology at a Glance. 2nd ed. Malden, MA: Blackwell Science Ltd; 2014.

[36] Georgeson MA , Wallis SA. Binocular fusion, suppression and diplopia for blurred edges. Ophthalmic Physiol Opt. 2014;34(2):163–185. 24476421 10.1111/opo.12108 PMC4312971

[37] Clark U . Visual tracking. In: Kreutzer J, DeLuca J, Caplan B, eds. Encyclopedia of Clinical Neuropsychology. Cham, Switzerland: Springer International Publishing; 2018:1–3.

[38] NHS. *Diagnosis: Short-Sightedness (Myopia)*. 2018. https://www.nhs.uk/conditions/short-sightedness/diagnosis/. Accessed May 17, 2021.

[39] Wilson LB , Melia M, Kraker RT, et al. Accuracy of autorefraction in children: a report by the American Academy of Ophthalmology. Ophthalmology. 2020;127(9):1259–1267. 32317177 10.1016/j.ophtha.2020.03.004

[40] Zammit S , Odd D, Horwood J, et al. Investigating whether adverse prenatal and perinatal events are associated with non-clinical psychotic symptoms at age 12 years in the ALSPAC birth cohort. Psychol Med. 2009;39(9):1457–1467. 19215630 10.1017/S0033291708005126

[41] Bouthry E , Picone O, Hamdi G, et al. Rubella and pregnancy: diagnosis, management and outcomes. Prenat Diagn. 2014;34(13):1246–1253. 25066688 10.1002/pd.4467

[42] Kinney DK , Teixeira P, Hsu D, et al. Relation of schizophrenia prevalence to latitude, climate, fish consumption, infant mortality, and skin color: a role for prenatal vitamin d deficiency and infections? Schizophr Bull. 2009;35(3):582–595. 19357239 10.1093/schbul/sbp023 PMC2669590

[43] Yazar S , Hewitt AW, Black LJ, et al. Myopia is associated with lower vitamin D status in young adults. Invest Ophthalmol Vis Sci. 2014;55(7):4552–4559. 24970253 10.1167/iovs.14-14589

[44] Cooper SA , Smiley E, Morrison J, et al. Psychosis and adults with intellectual disabilities. Prevalence, incidence, and related factors. Soc Psychiatry Psychiatr Epidemiol. 2007;42(7):530–536. 17502974 10.1007/s00127-007-0197-9

[45] Warburg M . Visual impairment in adult people with intellectual disability: literature review. J Intellect Disabil Res. 2001;45(Pt 5):424–438. 11679048 10.1046/j.1365-2788.2001.00348.x

[46] Davies C , Segre G, Estradé A, et al. Prenatal and perinatal risk and protective factors for psychosis: a systematic review and meta-analysis. Lancet Psychiatry. 2020;7(5):399–410. 32220288 10.1016/S2215-0366(20)30057-2

[47] Yu B , Dai L, Chen J, et al. Prenatal and neonatal factors for the development of childhood visual impairment in primary and middle school students: a cross-sectional survey in Guangzhou, China. BMJ Open. 2020;10(9):e032721. 10.1136/bmjopen-2019-032721 PMC7482504 32912936

[48] Mountjoy E , Davies NM, Plotnikov D, et al. Education and myopia: assessing the direction of causality by Mendelian randomisation. BMJ. 2018;361:k2022. 29875094 10.1136/bmj.k2022 PMC5987847

[49] Fusar-Poli P , Tantardini M, De Simone S, et al. Deconstructing vulnerability for psychosis: meta-analysis of environmental risk factors for psychosis in subjects at ultra high-risk. Eur Psychiatry. 2017;40:65–75. 27992836 10.1016/j.eurpsy.2016.09.003

[50] Goodman R . Psychometric properties of the strengths and difficulties questionnaire. J Am Acad Child Adolesc Psychiatry. 2001;40(11):1337–1345. 11699809 10.1097/00004583-200111000-00015

[51] Cox JL , Holden JM, Sagovsky R. Detection of postnatal depression: development of the 10-item Edinburgh Postnatal Depression Scale. Br J Psychiatry 1987;150(6):782–786. 3651732 10.1192/bjp.150.6.782

[52] Sharp C , Goodyer IM, Croudace TJ. The Short Mood and Feelings Questionnaire (SMFQ): a unidimensional item response theory and categorical data factor analysis of self-report ratings from a community sample of 7- through 11-year-old children. J Abnorm Child Psychol. 2006;34(3):365–377. 10.1007/s10802-006-9027-x 16649000

[53] Stochl J , Khandaker GM, Lewis G, et al. Mood, anxiety and psychotic phenomena measure a common psychopathological factor. Psychol Med. 2015;45(7):1483–1493. 25394403 10.1017/S003329171400261X

[54] StataCorp . 2019. Stata Statistical Software: Release 16. College Station, TX: StataCorp LLC.

[55] White IR , Royston P, Wood AM. Multiple imputation using chained equations: issues and guidance for practice. Stat Med. 2011;30(4):377–399. 21225900 10.1002/sim.4067

[56] Chaplin AB , Jones PB, Khandaker GM. Association between common early-childhood infection and subsequent depressive symptoms and psychotic experiences in adolescence: a population-based longitudinal birth cohort study. Psychol Med. 2020:1–11. 10.1017/S0033291720004080 PMC9386436 33183379

[57] Perneger TV . What’s wrong with Bonferroni adjustments. BMJ. 1998;316(7139):1236–1238. 9553006 10.1136/bmj.316.7139.1236 PMC1112991

[58] Pang L . Hallucinations experienced by visually impaired: Charles Bonnet syndrome. Optom Vis Sci. 2016;93(12):1466–1478. 27529611 10.1097/OPX.0000000000000959 PMC5131689

[59] Jurišić D , Ćavar I, Sesar A, Sesar I, Vukojević J, Ćurković M. New insights into schizophrenia: a look at the eye and related structures. Psychiatr Danub. 2020;32(1):60–69. 32303031 10.24869/psyd.2020.60

[60] Easty DL , Ferris J. Basic Sciences in Ophthalmology: A Self Assessment Text. UK: Wiley; 1998.

[61] City of Avon. *About*. 2021. https://www.cityofavon.org/demographics. Accessed June 18, 2021.

[62] Cornish RP , Macleod J, Carpenter JR, Tilling K. Multiple imputation using linked proxy outcome data resulted in important bias reduction and efficiency gains: a simulation study. Emerg Themes Epidemiol. 2017;14:14. 29270206 10.1186/s12982-017-0068-0 PMC5735815

[63] Keane BP , Kastner S, Paterno D, Silverstein SM. Is 20/20 vision good enough? Visual acuity differences within the normal range predict contour element detection and integration. Psychon Bull Rev. 2015;22(1):121–127. 24845876 10.3758/s13423-014-0647-9 PMC4240750

[64] Dalman C , Thomas HV, David AS, Gentz J, Lewis G, Allebeck P. Signs of asphyxia at birth and risk of schizophrenia: population-based case–control study. Br J Psychiatry. 2001;179(5):403–408. 11689395 10.1192/bjp.179.5.403

[65] Croft J , Heron J, Teufel C, et al. Association of trauma type, age of exposure, and frequency in childhood and adolescence with psychotic experiences in early adulthood. JAMA Psychiatry. 2019;76(1):79–86. 30477014 10.1001/jamapsychiatry.2018.3155 PMC6490231

[66] Silverstein SM , Fradkin SI, Demmin DL. Schizophrenia and the retina: towards a 2020 perspective. Schizophr Res. 2020;219:84–94. 31708400 10.1016/j.schres.2019.09.016 PMC7202990

[67] Hébert M , Gagné AM, Paradis ME, et al. Retinal response to light in young nonaffected offspring at high genetic risk of neuropsychiatric brain disorders. Biol Psychiatry. 2010;67(3):270–274. 19833322 10.1016/j.biopsych.2009.08.016

