# Supplementary material for: Association Between Childhood Visual Acuity and Late Adolescent Psychotic Experiences: A Prospective Birth Cohort Study
Source: Schizophr Bull. 2021 Oct 8;48(2):325–34. doi: 10.1093/schbul/sbab121 (PMC8886579; doi:10.1093/schbul/sbab121)
Supplement: sbab121_suppl_Supplementary_Tables [file sbab121_suppl_supplementary_tables.docx]

Supplementary table 1: Characteristics of participants with and without missing analytic data from sample with primary exposure data at age 7 and at least one short Mood and Feelings Questionnaire score

| **Characteristic** | **Sample with missing Data**  **N (%)** | **Sample without missing data**  **N (%)** |
| --- | --- | --- |
| Total | 3,637 (54.4) | 3,049 (45.6) |
| Male | 1,944 (53.6 | 1,361 (44.6) |
| Mother’s socioeconomic status based on occupation | | |
| Professional | 65 (2.6) | 177 (5.8) |
| Managerial and technical | 697 (28.2) | 1,102 (36.1) |
| Skilled non-manual | 1,106 (44.7) | 1,254 (41.1) |
| Skilled manual | 99 (4.0) | 101 (3.3) |
| Partly skilled | 413 (16.7) | 369 (12.1) |
| Unskilled | 92 (3.7) | 46 (1.5) |
| Maternal educational level | | |
| CSE | 515 (16.7) | 235 (7.7) |
| Vocational | 304 (9.8) | 208 (6.8) |
| O level | 1,143 (37.0) | 1,057 (34.7) |
| A level | 752 (24.3) | 914 (30.0) |
| Degree | 377 (12.2) | 635 (20.8) |
| Mother’s partner’s educational level | | |
| CSE | 671 (22.2) | 450 (14.8) |
| Vocational | 256 (8.7) | 210 (6.9) |
| O level | 693 (23.6) | 668 (21.9) |
| A level | 792 (26.9) | 898 (29.5) |
| Degree | 528 (18.0) | 823 (27.0) |
| Infection during 1^st^ trimester of pregnancy | 661 (23.6) | 716 (23.5) |
| Maternal smoking in pregnancy | 722 (22.6) | 408 (13.4) |
| Mother’s parity in pregnancy | Median: 0  IQR: 0-1 | Median: 0  IQR: 0-1 |
| IQ aged 8 | Mean: 102.5  SD: 15.8 | Mean: 108.3  SD: 15.7 |
| SDQ score aged 81 months | Median: 7  IQR: 4-11 | Median: 6  IQR: 4-9 |
| Maternal EPDS score in Pregnancy | Median: 6  IQR: 3-10 | Median: 6  IQR: 3-9 |
| Maternal vitamin D consumption in pregnancy in micrograms | Median: 3.4  IQR: 2.3 – 5.2 | Median: 3.6  IQR: 2.6 – 5.6 |
| LogMAR score aged 7 | Mean: -0.06  SD: 0.08 | Mean: -0.06  SD: 0.07 |
| LogMAR score aged 11 | Mean: -0.14  SD: 0.09 | Mean: -0.15  SD: 0.08 |
| Needed glasses aged 7 | 381 (10.5) | 309 (10.2) |
| Needed glasses aged 11 | 485 (18.9) | 498 (17.6) |
| Visual Impairment (LogMAR > 0 or needing glasses) at age 7 | 700 (19.3) | 551 (18.1) |
| Visual Impairment (LogMAR > 0 or needing glasses) at age 11 | 505 (20.1) | 520 (18.7) |
| Manifest Strabismus aged 7 | 67 (1.8) | 64 (2.1) |
| Abnormal Prism test aged 7 | 358 (9.9) | 336 (11.1) |
| History of eyepatch aged 7 | 108 (3.0) | 106 (3.5) |
| Abnormal Worth’s Four Dots test aged 7 | 117 (4.1) | 76 (3.3) |
| Impaired near vision aged 7 | 150 (4.1) | 106 (3.5) |
| Abnormal saccadic eye movements aged 7 | 173 (5.4) | 152 (5.8) |
| Abnormal smooth pursuit eye movements aged 7 | 239 (6.6) | 237 (7.8) |
| Scored positive on PLIKSi aged 17 | 75 (8.0) | 184 (7.1) |
| Scored positive on PLIKSi aged 24 | 85 (11.7) | 204 (9.5) |
| Scored positive on PLIKSi at either age | 141 (12.7) | 336 (11.0) |

N = Number, CSE = Certificate of Secondary Education, IQ = Intelligence Quotient, LogMAR= Logarithm of Minimal Angle of Resolution, where 0 = “normal” vision, <0 = “ better than normal” vision, and >0 = reduced vision; EPDS = Edinburgh Postnatal Depression Scale; SDQ = Strengths and Difficulties Questionnaire; PLIKSi = Psychotic-like Experiences Symptoms Interview.

Supplementary Table 2: Odds of Scoring Positive on Psychotic-Like Symptoms Interview (PLIKSi) According to Eyesight Variables Excluding Participants who reported Visual Hallucinations in complete case sample

| **Exposure** | **N** | **OR (95% CI)** | **P-value** | **AOR (95% CI) ˪** | **P-value** |
| --- | --- | --- | --- | --- | --- |
| **Outcome: Positive result on PLIKSi aged 24 or aged 17** | | | | | |
| Best Corrected Visual Acuity aged 7 Ɨ | 3,005 | 1.44 (1.12 – 1.85) | 0.004 | 1.41 (1.10 – 1.82) | 0.008* |
| Best Corrected Visual Acuity aged 11 Ɨ | 3,025 | 1.29 (1.03 – 1.60) | 0.025 | 1.25 (0.99 – 1.57) | 0.051 |
| Difference in Acuity between eyes aged 7 Ɨ | 3,005 | 1.00 (0.74 – 1.35) | 0.998 | 0.97 (0.72 – 1.32) | 0.863 |
| Difference in Acuity between eyes aged 11 Ɨ | 3,025 | 1.04 (0.68 – 1.59) | 0.847 | 1.06 (0.69 – 1.62) | 0.794 |
| Child needed glasses aged 7 | 3,324 | 1.42 (0.84 – 2.41) | 0.186 | 1.31 (0.78 – 2.22) | 0.306 |
| Child needed glasses aged 11 | 3,089 | 1.62 (1.02 – 2.58) | 0.040 | 1.61 (1.01 – 2.55) | 0.044* |
| Normal vision with glasses or subnormal vision aged 7 ˩ | 2,999 | 1.58 (0.99 – 2.50) | 0.050 | 1.49 (0.94 – 2.37) | 0.090 |
| Normal vision with glasses or subnormal vision aged 11 ˩ | 3,024 | 1.78 (1.12 – 2.83) | 0.014 | 1.72 (1.09 – 2.73) | <0.001* |
| Manifest strabismus aged 7 | 3,326 | 0.21 (0.04 – 1.16) | 0.073 | 0.18 (0.31 – 1.01) | 0.051 |
| History of eyepatch aged 7 | 3,331 | 0.59 (0.21 – 1.63) | 0.310 | 0.51 (0.18 – 1.43) | 0.199 |
| Abnormal prism test aged 7 | 3,318 | 0.93 (0.54 – 1.61) | 0.793 | 0.86 (0.50 – 1.50) | 0.602 |
| Abnormal Worth Four Dots Test aged 7 | 2,517 | 0.63 (0.20 – 1.99) | 0.428 | 0.56 (0.18 – 1.76) | 0.322 |
| Impaired near vision aged 7 | 3,312 | 0.56 (0.20 – 1.57) | 0.270 | 0.51 (0.18 – 1.43) | 0.201 |
| Abnormal saccadic eye movements aged 7 | 2,881 | 0.47 (0.19 – 1.15) | 0.099 | 0.40 (0.16 – 0.99) | 0.047* |
| Abnormal pursuit eye movements aged 7 | 3,324 | 0.43 (0.20 – 0.91) | 0.028* | 0.44 (0.21 – 0.93) | 0.032* |

N = Number of individuals in analysis; OR = Odds Ratio; 95% CI = 95% Confidence Interval; AOR = Adjusted Odds Ratio; Ɨ per 0.1 point deterioration; ˩ = relative to group with normal vision without glasses.

˪ = Adjusted for sex; mother’s socioeconomic status; educational level of mother and mother’s partner; maternal smoking during pregnancy; perinatal infection during first trimester; parity of mother during pregnancy; mother’s reported vitamin D intake during pregnancy; Strengths and Difficulties Questionnaire (SDQ) score aged 81 months; and maternal Edinburgh Postnatal Depression Scale (EPDS) score in pregnancy.

At age 11, this was further adjusted for IQ aged 8.

* indicates p<0.05
